# Water Stable Isotopes in Ecohydrological Field Research: Comparison Between *In Situ* and Destructive Monitoring Methods to Determine Soil Water Isotopic Signatures

**DOI:** 10.3389/fpls.2020.00387

**Published:** 2020-04-14

**Authors:** Angelika Kübert, Sinikka Paulus, Adrian Dahlmann, Christiane Werner, Youri Rothfuss, Natalie Orlowski, Maren Dubbert

**Affiliations:** ^1^Ecosystem Physiology, University of Freiburg, Freiburg, Germany; ^2^Department of Biogeochemical Integration, Max Planck Institute for Biogeochemistry, Jena, Germany; ^3^Institute of Bio- and Geosciences, Agrosphere Institute (IBG-3), Jülich, Germany; ^4^Chair of Hydrology, University of Freiburg, Freiburg, Germany

**Keywords:** *in situ* soil water vapor sampling technique, cryogenic vacuum extraction, centrifugation, water stable isotopes, soil water, ecohydrology

## Abstract

Ecohydrological isotope based field research is often constrained by a lack of temporally explicit soil water data, usually related to the choice of destructive sampling in the field and subsequent analysis in the laboratory. New techniques based on gas permeable membranes allow to sample soil water vapor *in situ* and infer soil liquid water isotopic signatures. Here, a membrane-based *in situ* soil water vapor sampling method was tested at a grassland site in Freiburg, Germany. It was further compared with two commonly used destructive sampling approaches for determination of soil liquid water isotopic signatures: cryogenic vacuum extraction and centrifugation. All methods were tested under semi-controlled field conditions, conducting an experiment with dry-wet cycling and two isotopically different labeling irrigation waters. We found mean absolute differences between cryogenic vacuum extraction and *in situ* vapor measurements of 0.3–14.2‰ (δ^18^O) and 0.4–152.2‰ (δ^2^H) for soil liquid water. The smallest differences were found under natural abundance conditions of ^2^H and ^18^O, the strongest differences were observed after irrigation with labeled waters. Labeling strongly increased the isotopic variation in soil water: Mean soil water isotopic signatures derived by cryogenic vacuum extraction were -11.6 ± 10.9‰ (δ^18^O) and +61.9 ± 266.3‰ (δ^2^H). The *in situ* soil water vapor method showed isotopic signatures of -12.5 ± 9.4‰ (δ^18^O) and +169.3 ± 261.5‰ (δ^2^H). Centrifugation was unsuccessful for soil samples due to low water recovery rates. It is therefore not recommended. Our study highlights that the *in situ* soil water vapor method captures the temporal dynamics in the isotopic signature of soil water well while the destructive approach also includes the natural lateral isotopic heterogeneity. The different advantages and limitations of the three methods regarding setup, handling and costs are discussed. The choice of method should not only consider prevailing environmental conditions but the experimental design and goal. We see a very promising tool in the *in situ* soil water vapor method, capturing both temporal developments and spatial variability of soil water processes.

## Introduction

Water stable isotopes (^2^H and ^18^O) are considered as ideal tracers to study water fluxes within the soil-plant-atmosphere continuum (Rothfuss and Javaux, 2017; Dubbert and Werner, 2019). In order to retrieve the water isotopic signature of soil water, soil material is predominantly sampled destructively and containing water extracted in the laboratory (Orlowski et al., 2016b). The most commonly used methods are cryogenic vacuum extraction, centrifugation or high pressure mechanical squeezing, but also new methods such as microwave extraction and direct vapor equilibration have been developed and compared (Orlowski et al., 2016b). These destructive methods, however, are very limited in terms of data point sampling and therewith restrict the study of ecohydrological processes. Berry et al. (2018) addressed the methodological state of the art in stable isotope ecohydrology as “shotgun” or “snapshot methods”, referring to the lack of continuous measurements. Laboratory based water extraction methods are currently highly debated due to their inaccuracy and non-comparability of the obtained isotope results (Orlowski et al., 2019). Issues observed with laboratory based methods are mainly due to interferences with soil texture, water contents, interactions with cations, and the different pore spaces that may or may not be extracted via the different approaches (Meißner et al., 2014; Oerter et al., 2014; Orlowski et al., 2016b, 2018).

Several authors have addressed the lack of methodological consistency in water stable isotope research and called for an improvement of spatial and temporal resolution of soil pore water, vapor fluxes and plant transpiration measurements (Vereecken et al., 2015; Volkmann et al., 2016b; Berry et al., 2018; Dubbert and Werner, 2019). Techniques based on laser spectroscopy allow to measure online and *in situ* water vapor isotopic signature at high precision and frequency (Helliker and Noone, 2010; Werner et al., 2012). Combining laser spectroscopy with other techniques also allows for measuring different water compartments and fluxes at high temporal resolution, such as soil pore water (e.g., Rothfuss et al., 2013; Volkmann and Weiler, 2014; Gaj et al., 2016), xylem water (Volkmann et al., 2016a) or transpiration (Wang et al., 2012; Simonin et al., 2013; Dubbert et al., 2017). Microporous polypropylene membranes, for instance, enabled the sampling of soil water vapor isotope compositions *in situ* and non-destructively once installed (Rothfuss et al., 2013; Volkmann and Weiler, 2014; Gaj et al., 2016).

So far, this *in situ* soil water vapor technique has been mainly applied for continuous measurements under controlled laboratory conditions (Rothfuss et al., 2015; Quade et al., 2018), or on short timescales in the field (maximum 11 consecutive days) (Gaj et al., 2016; Volkmann et al., 2016b) and only few times during long-term experiments under field conditions (Oerter et al., 2017; Quade et al., 2019). Despite few applications, the potential of *in situ* long-term monitoring experiments has been addressed by numerous studies and reviews within the field of ecohydrology (Sprenger et al., 2015, 2019; Berry et al., 2018; Penna et al., 2018; Stumpp et al., 2018). With continuous *in situ* isotope measurements of soil and xylem water vapor, Volkmann et al. (2016a), for instance, were able to show how different tree species under drought varied in their reaction toward an artificial precipitation pulse. Their results showed that the response was visible within one to four hours after the irrigation event and that the travel times were species specific. More experiments of this type are needed in order to evaluate the relevance of the observed patterns for different soil and vegetation types and over longer time scales. In particular, high uncertainties exist concerning the reaction of ecosystems in dry conditions with low soil water availability when plants need to optimize cost and benefit between transpiration and biomass production (Schymanski et al., 2008). An enhanced understanding of these processes would open the path to improve physically based modeling approaches of actual and future water fluxes of different ecohydrological compartments (Rothfuss and Javaux, 2017). Furthermore, it could help to develop management and conservation strategies to sustain water resources as well as augment eco- and agricultural system resilience (Mahindawansha et al., 2018; Penna et al., 2018). In particular, the management and conservation of grasslands are of economic and ecological interest. They provide important ecosystem services such as biodiversity conservation and carbon storage (White et al., 2000; Millennium Ecosystem Assessment, 2005; Stoate et al., 2009; Schnyder et al., 2010; Maestre et al., 2012). Global change induced threats such, as extreme drought or heavy rainfall can, however, substantially threat their stable functioning and hence the provided ecosystem services (Habel et al., 2013; IPBES, 2018).

The central objective of our study was the comparison between *in situ* and destructive estimates of soil water isotopic signatures observed in a semi-controlled field experiment at a grassland site (Freiburg, Germany). The applied *in situ* method was a membrane based monitoring method developed by Rothfuss et al. (2013) for soil water vapor. We compared the *in situ* water vapor method for soil water with the two commonly used destructive soil water sampling approaches – cryogenic vacuum extraction and centrifugation. We characterized the temporal dynamics of the isotopic signature of soil water in different soil depths.

We tested the three approaches under (semi-controlled) field conditions by conducting an experiment with dry-wet cycling and isotopically labeled water in order to test their application (i) under varying micrometeorological conditions, (ii) for different soil water contents, and (iii) for different ranges of isotopic signature (natural and labeled abundance). For this purpose, we artificially created water limited conditions (14 days) under isotopic natural abundance conditions, and subsequently applied two severe rain pulses (20 mm per 70 min and 35 mm per 90 min), each followed by a period of drought (12 and 18 days). The two pulses differed in their isotopic signatures, one pulse being strongly depleted in both isotopologues (^18^O and ^2^H) and one pulse being heavily deuterated relative to natural abundance.

The different advantages and limitations of the three methods are discussed in detail (including not only their accuracy but also related expenses of time and costs for setup and use) in order to enhance future consideration about methodological approaches to address ecohydrological and soil water processes related questions.

## Materials and Methods

The measured or inferred values of water hydrogen and oxygen stable isotopic ratios are reported relative to the values of the Vienna Standard Mean Ocean Water and expressed as isotopic signatures (respectively, δ^2^H and δ^18^O) in per mill (‰) (Gonfiantini, 1978). All isotopic analysis was performed with a cavity ring-down laser spectroscope (L2130-i; Picarro, Santa Clara, United States).

### Study Site

The study was performed at the experimental research site Freiburg Flugplatz in South West Germany at 238 m a.s.l. (48°1′22′′N; 7°49′57′′O). The site represents a temperate, perennial grassland with ruderal vegetation. Mean annual temperature is 11.4°C and mean annual rainfall is 662.1 mm (reference period 1988–2017, Deutscher Wetterdienst [DWD], 2018). The soil is classified as a skeleton-rich Anthrosol on former fluvial deposition and displays pronounced differences between soil horizons. Top layer is brown earth (0–10 cm), followed by a sandy, medium grain gravel layer (10–35 cm) with consolidated clay beneath 40 cm depth. Between 0 and 40 cm depth, soil texture consisted of 37.8% sand, 48.6% silt, and 13.7% clay. Porosities in the soil ranged between 57% in the topsoil and 36% in the gravel layer (K. Kühnhammer, personal communication). Dominant species during the time of our experiment were *Agrostis tenuis* L*., Carex hirta* L. and *C. jacea.*

### Generating Varying Soil Water Contents and Isotopic Signatures

Different water contents and isotopic signature values were imposed in the soil profile by excluding natural precipitation and simulating irrigation events. For this, we installed a plot of 4 m × 3 m inner size which could be covered up by a transparent rainout shelter in 145 to 210 cm height above the canopy. These shelters included a buffering zone of approximately 100 cm at each side to prevent subsurface lateral flow from adjacent soil. First measurements started on June 15, 2018. Natural precipitation was excluded from the plot after June 7, 2018. Shelters were set up exclusively before forecasted rain events.

Irrigation events took place on June 20 and July 2 in the late evening (22:00–23:10; 22:00–23.30 CET, respectively). The water of the first irrigation event (referred to as “label 1”) was labeled with δ^18^O = −56.9 ± 0.8‰ and δ^2^H = −181.7 ± 5.2‰ and simulated a rain event of 20 mm per 70 min. The water of the second irrigation event (referred to as “label 2”, deuterated) was labeled with δ^18^O = −10.3 ± 0.2‰ and δ^2^H = +396.0 ± 16.4‰ and simulated a rain event of 35 mm per 90 min. The chosen irrigation amounts correspond to rain events classified as “severe precipitation events” and “storm warning”, respectively, according to the classification of the German Meteorological Service DWD. Irrigation was performed at night to minimize evaporative losses during tracer application. Irrigation was applied by moving, 1 m above the plot in regular intervals, a 3 m long tubing, perforated each centimeter with a 1/16″ diameter tube.

In the following, the three experimental phases will be distinguished according to the irrigation events, i.e., between natural abundance, label 1 and label 2. The time is expressed in Day after Labeling (after label 1, DaL), i.e., natural abundance (before labeling, DaL = 0), label 1 (DaL 1–12) and label 2 (DaL 12–30). During all phases, natural precipitation was excluded by setting up a shelter before forecasted events.

### Micrometeorological Data

Environmental data were collected throughout the whole experiment. Photosynthetic photon flux density (S-LIA-M003, Onset, Bourne, MA, United States), air temperature and relative humidity (S-THB-M008, Onset, Bourne, MA, United States) were measured in a distance of 10 m from the experimental site in 1 m height above ground and logged as five-minute averages (HOBOH21-002 U30, Onset, Bourne, MA, United States). Natural bulk precipitation was sampled by a self-built precipitation collector. The collector consisted of glass funnel (diameter 80 cm^2^, at 1 m height above ground level) which was connected by an insulated PTFE (Polytetrafluoroethylene) tube to a glass bottle, buried in the ground. In the funnel, a coarse filter mesh was used to prevent contamination as much as possible. Bulk water samples were collected after each rain event, filtered to remove organic compounds (glass fiber, retention capacity 1–2 μm, KC98.1, Roth, Karlsruhe, Germany) and their isotopic signatures determined.

### Soil Water Content and Soil Temperature Measurements

Volumetric soil water contents (10 HS Decagon Devices, WA, United States) and soil temperatures (T108, Campbell Scientific, Logan, UT, United States) were monitored in −2, −5, −20 and −40 cm depth (*n* = 3). Sensors were set up on March 26–12 weeks before measurement start (June 15) – in order to minimize the impact on the vegetation cover. A 60 cm × 50 cm hole was excavated, and sensors were installed in the wall of the undisturbed soil. Soil excavation material was carefully emplaced trying to imitate original stratifications and eliminate the formation of preferential flow pathways. Sensor data was stored as five-minute averages with a data logger (CR1000, Campbell Scientific, Logan, UT, United States). The soil water content sensors’ readings were calibrated against values obtained gravimetrically from destructive sampling. Soil gravimetric water content was converted to volumetric water content by taking the soil dry bulk density (1.4 ± 0.2 g/cm^3^) into account.

### *In Situ* Isotopic Measurements of Soil Pore Water

#### Setup and Data Collection

**FIGURE 1:**
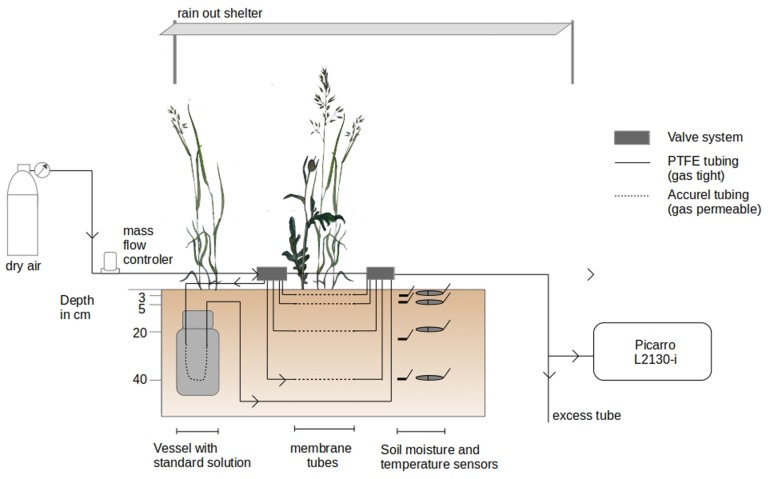
Experimental setup of the *in situ* soil water vapor measurements. Soil water vapor was sampled across the soil profile from the vicinity of a series of gas permeable polypropylene tubing. The *in situ* soil water vapor sampling method was coupled to a laser spectroscope (Picarro L2130-i) for isotope measurements.

The isotopic signature (δ_S_v_, i.e., δ^2^H and δ^18^O in ‰) and mixing ratio of soil water vapor (MR, i.e., the ratio of the mass of soil water vapor to the mass of dry air in a given volume in ppm) were measured non-destructively on a daily basis between 8:30 AM and 19:00 CET in −2, −5, −20 and −40 cm depth (*n* = 3), at the same depth as soil water content and temperature sensors were installed (Figure 1). It was done by following the method of Rothfuss et al. (2013), i.e., by sampling the water vapor intruding a piece of gas permeable (GP) microporous polypropylene tubes (Accurel GP V8/2HF, 3M, Germany; 0.155 cm wall thickness, 0.55 cm i.d., 0.86 cm o.d.) inserted in the soil. The water vapor contained in the tube eventually reaches isotopic thermodynamic equilibrium, i.e., has an isotopic signature function of that of liquid water and soil temperature (Majoube, 1971; Horita and Wesolowski, 1994). The soil water vapor is then flushed at a flow with dry synthetic air and directed toward the laser spectroscope for online determination of its isotopic signature.

GP tubes were installed on March 26 at the three sides of the excavated hole. During installation of the setup, two belowground connections of GP tubes were damaged (replicate 3, depths −2 and −40 cm) and, therefore, excluded from the experiment. The final setup comprised a total of 10 observation depths for δ_S_v_. The GP tubes were of 20 cm length (54.0 cm^2^ outer, 34.6 cm^2^ inner surface area, 4.8 cm^3^ inner volume) and extended at both ends with insulated PFA (Perfluoroalkoxy alkanes) and PTFE (Polytetrafluoroethylene) tubing systems (1/4″ and 1/8″). Dry synthetic air (Messer, Bad Soden, Germany) flow introduced to the tubes’ inlet could be distributed via electric valve manifolds (Oxygen Clean Manifold, Clippard Instruments, OH, United States) to a desired tube section from which the respective water vapor sample was directed to the laser spectroscope. Air flow was controlled by a Mass Flow Controller (MFC; model FC-260, Tylan, Torrance, CA, United States with a read-out box RO-7010).

δ_S_v_ of each GP tube was determined on a daily basis between 8:30 AM and 19:00 CET by directing synthetic dry air into the GP tubes at an average flow rate of 110 ml min^–1^. Before measurements, valve boxes and gas lines (excluding the GP soil gas sampling tubes) were flushed with synthetic dry air via bypass lines at a flow rate of 300 to 700 ml min^–1^ to evacuate water vapor or condensed droplets of previous measurements in the sampling system. In wet measurement periods (relative humidity >80% and/or soil volumetric water content in 3 cm > 15%), each GP tube was flushed for five minutes at a flow rate of 300 ml min^–1^. During drier time periods, no flushing of the GP tubes was necessary. Additionally, before/after each GP tube measurement, the tube system was flushed for five minutes via bypass lines. Steady values in MR and δ_S_v_ were, generally, reached after 10–20 min of sampling. Soil water vapor was sequentially sampled from the GP tubes in the following order for each profile: −20, −2, −40, and −5 cm depth. In total, δ_S_v_ was measured for each GP tube once a day during the experiment except on weekends.

For calibration of the raw δ_S_v_ values, water vapor was sampled sequentially from three custom-built soil standards (one isotopically ‘light’ standard STD_L_, one ‘medium’ standard STD_M_, and one heavy standard STD_H_) once a day following the above described method. For this, three laboratory containers equipped with gas permeable GP tubes were filled with quartz sand (0.3–0.8 mm) and saturated (i.e., approx. 35 % vol. SWC) with water of known isotopic signature (STD_L_: δ^18^O = −78.8 ± 0.04‰; δ^2^H = −263.3 ± 0.71‰; STD_M_: δ^18^O = −9.3 ± 0.07‰; δ^2^H = −65.3 ± 0.32‰; STD_H_: δ^18^O = −9.3 ± 0.2‰; δ^2^H = +865.0 ± 0.21‰). The STD_L_ and STD_H_ were measured last in order to prevent carry-over (memory) effects in the laser spectroscope. All standards were buried, together with a temperature sensor (T108, Campbell Scientific, Logan, UT, United States), in −60 cm depth in the soil at a distance of approximately 4 m from the center of the experimental plot. Insulating material was placed between standard vessel lids and topsoil material to keep changes of temperature of standards as small as possible.

#### Data Processing

Assuming thermodynamic equilibrium, collected data on δ_S_v_ were converted to liquid soil isotopic signatures δ_S_l_ after Majoube (1971) (Eq. 1 and 2) using the soil temperature of the respective soil depth. For the soil water vapor standards, the temperature of the adjacent buried sensor was used.

(1) $$\delta_{\text{S\_l}}=\left(\delta_{\text{S\_v}}+1\right)\times \alpha^{+}-1$$

(2) $$\text{ln}\,\alpha^{+}=\text{a}\,\frac{10^{6}}{T^{2}}+\text{b}\,\frac{10^{3}}{\text{T}}+\text{c}$$

With α^+^ being the equilibrium fractionation factor, T the reaction temperature in Kelvin. α^+^ was calculated based on the coefficients a, b, c from the experimental results of Majoube (1971) (i.e., for δ_s_l_ of ^2^H: a = 24.844, b = −76.248 and c = 52.612; for δ_s_l_ of ^18^O: a = 1.137, b = −0.4156, c = −2.0667).

To check for dependencies between MR and δs_l, the linear relationship between MR and the deviation of the measured δs_l value from the true δs_l value was determined based on the measurements of the soil standard vessels (Schmidt et al., 2010). No significant relationships were found and, therefore, no corrections applied (δ^2^H: *p* = 0.15, R^2^ = 0.11; δ^18^O: *p* = 0.8, R^2^ = −0.04, Supplementary Figure S1).

Subsequently, plateaus of steady values in MR, δ^2^H, and δ^18^O (liquid) were determined by moving averages of the coefficients of variation (cv, 3 min period). Intervals of steady values were identified by finding the time period (x) when the sum of the cvs (MR, δ^2^H, and δ^18^O) was the smallest. Finally, isotopic signatures of selected intervals were averaged and calibrated against the ones of STD_M_ by drift correction. STD_L_ and STD_H_ were not used for calibration due to continuous problems with carry over effects.

### Soil Destructive Sampling for Isotope Analysis

For comparison with the *in situ* method, soil samples were destructively taken every three days (13 days in total). All samples were taken within distances of maximum 1.80 m from the corners of the *in situ* measurement setup (Figure 1). Soil cores were collected with a soil auger (core diameter 20 mm, Pürckhauer, ecoTech Umwelt-Meßsysteme GmbH, Bonn, Germany) and stratified into portions of 0–3 cm, 3–8 cm, 18–23 cm, and 38–43 cm depth to correspond to the positioning of the *in situ* GP tubes (−2, −5, −20, and −40 cm depth). For each soil depth, three replicates were sampled per day (*n* = 3), except for centrifugation of soil (*n* = 2). Moreover, sampling for centrifugation was stopped 11 days before the other methods since the water recovery rates of soil samples were low (see section 3.3). Soil samples were always taken between 14:30 and 15:00 CET. Immediately after sampling, samples were split into two subsamples: 1) gas-tight 10-ml septum-capped glass vials for cryogenic extraction and 2) gas-tight centrifuge tubes (20 ml Pall Macrosep Advance) and sealed with Parafilm (Bemis, Oshkosh, WI, United States). All samples were kept in a cool box during transportation and stored at −20°C until further analysis.

### Cryogenic Vacuum Extraction and Centrifugation

Soil destructive samples were extracted via: (1) cryogenic vacuum extraction, and (2) centrifugation. For cryogenic vacuum extraction, samples were heated up to approximately 95°C for 90 min under a vacuum of 0.1 mbar (XDS10 vacuum pump, Edwards, Burgess Hill, United Kingdom) and water vapor was trapped in liquid N_2_ traps (custom-built vacuum line, similar to Ehleringer and Dawson (1992); design of R. Siegwolf). For centrifugation, samples were defrosted in the gas-tight centrifuge tubes, sealed with Parafilm. After defrosting, they were spun for 1 h at 5000 rpm at a temperature of 40°C on a Rotina centrifuge [Rotina 48 R, Hettich, Tuttlingen, Germany, similar to Millar et al. (2018)]. After water extraction, liquid water samples were stored in thread glass vials (ND9, LLG, Meckenheim, DE) with closed lids at 4°C until isotope analysis. Isotope analysis was conducted with a V1102-i vaporization module coupled to a A0325 robotic autosampler (both accessories of the L2130-i laser spectroscope, Picarro Inc., Santa Clara, CA, United States) and then measured with the same laser spectroscope which was used in the field. Extracted water samples were calibrated against three laboratory standards (Newman et al., 2009). All laboratory standards and soil standards were calibrated against international standards: V-SMOW, SLAP, and GISP (IAEA, Vienna). Water vapor mixing ratios did not strongly vary (16,143 ± 1,402 ppmv), and, therefore, no correction was applied to the isotope measurement results (see section 2.5). In addition, isotope results of extracted water samples were post-processed with ChemCorrect^TM^ (Picarro Inc., Santa Clara, CA, United States) to detect organic contamination.

In order to quantify the effect of soil properties on cryogenic vacuum extraction, we fully dried soil samples from the field site (105°C for >48 h) and added water of known isotopic signature (spike test, Thielemann et al., 2019). Since water contents have shown to affect the soil water isotopic signature, this spike test was run for two different volumetric water contents (15% and 30%, *n* = 10, respectively). Soil samples were kept frozen and subsequently extracted cryogenically.

For both extraction methods, water recovery rates [%] were calculated on basis of the weight loss (mC-1 Laboratory LC 620, Sartorius AG, Göttingen, Germany) after oven drying (at 100°C for 48 h) of the extracted samples.

### Statistical Analysis and Evaluation

Statistical analysis was conducted in R [R Core Team (2017), version 3.6.0]. Results were presented as mean values ± 1 standard deviation (SD) if not stated differently.

Dual-isotope graphical representation was used to compare between *in situ* and destructive laboratory-based isotope measurements. Locally estimated scatterplot smoothing (loess) was applied to find significant differences between extraction methods (only for experimental phases label 1 and label 2). Only data of at least two replicates per date and depth were included in the analysis. Centrifugation soil data was excluded due to the small sample size. Methods were considered significantly different if the confidence intervals (95%) of their respective loess functions did not overlap [software package ggplot2 by Wickham (2016)]. This approach was chosen because of the small and unequal sample size of the different methods and the repetitive measurement character of the *in situ* soil water vapor method.

Based on the same data, boxplots were created (showing also the sample size of each method per experimental phase) and the mean and SD across experimental phases calculated (per date as well as over the whole phase; per depth and across all depths) including error propagation. Moreover, the difference between soil water isotopic signature mean values (over the experimental phase) before and after the labelling pulses was calculated to determine the effect of labelling pulses 1 and 2.

Linear regression analysis was employed to evaluate the relationship between water vapor mixing ratios and isotopic signatures obtained by the *in situ* soil water vapor sampling method and further to determine the local meteorological water line (LMWL, based on year 2017) of the field site. Before analysis, residual versus fitted plots and quantile-quantile plots based on the model were plotted to validate the assumptions of homogenous variance and normal distribution of residuals of linear regression analysis.

## Results

### Micrometeorological Conditions

**FIGURE 2:**
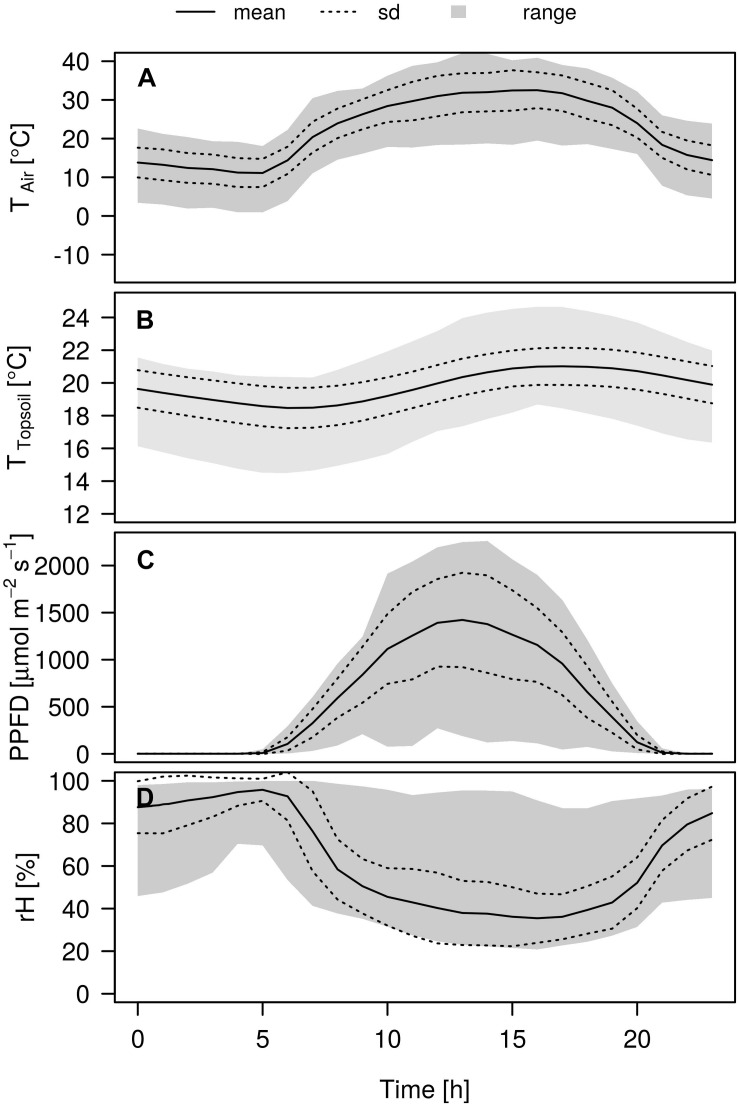
Variation in micrometeorological conditions during the day over the course of the experiment. **A** Air temperature T_Air_ [°C], **(B)** topsoil temperature T_Soil_ [°C], **(C)** photosynthetic photon flux density PPFD [μmol m^–2^ s^–1^], and **(D)** Relative humidity rH [%]. Mean (solid lines) ± 1 SD (dashed lines) and range (gray shaded).

Air temperature showed a strong amplitude during the experiment and ranged from 0.9 up to 43.3°C (Figure 2). The prevailing development of the mean air temperature during the day was characterized by a strong increase from 11.1°C at 5:00 CET, reaching a stable maximum at noon of around 31.2°C, before declining slowly toward the evening again. Topsoil temperatures (−2 cm depth) followed the same pattern, although the amplitude was less pronounced and approximately 2 h delayed from the air temperature, reaching its mean temperature maximum of 22.0°C around 18:00 CET. Daily temperature amplitudes were strongest in the topsoil and decreased with decreasing depth (data not shown). The photosynthetic photon flux density was highest at 13:00 CET reaching mean intensities of 1422.4 μmol m^–2^ s^–1^. Air relative humidity was inversely related to air temperature, reaching its mean daily minimum of 35.5% around 4 pm whereas maximum values of 94.7% could be observed around 5:00 CET in the morning.

### Soil Water Contents

**FIGURE 3:**
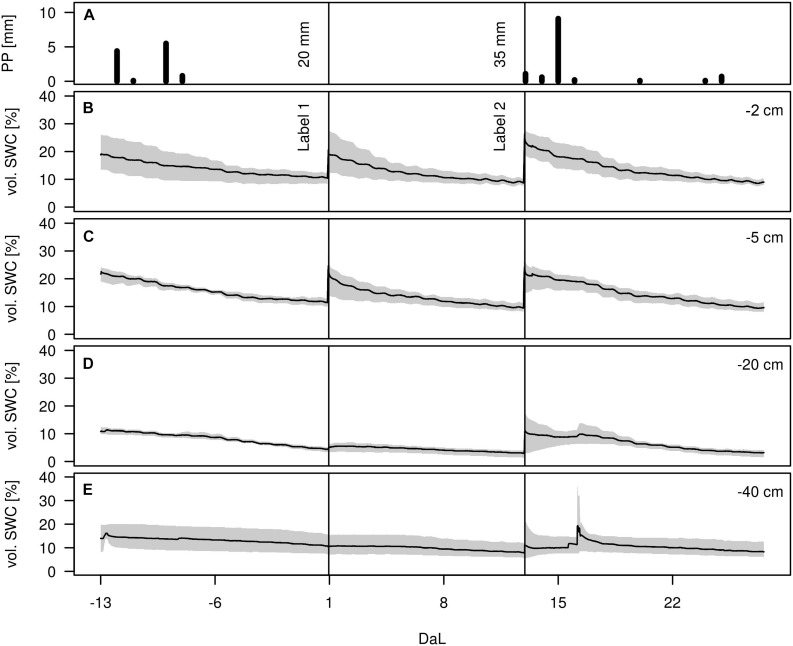
**(A)** Precipitation height [mm], **(B–E)** Volumetric soil water content (vol. SWC, in %) in −2, −5, −20, and −40 cm during the experiment (Day after Labelling DaL). Black lines indicate irrigation events: label 1 and label 2. Mean (solid range) and range (gray shaded), *n* = 3.

During the whole experiment, only few precipitation events occurred, with the strongest one on July 5, amounting to 9 mm (Figure 3). The effects of the rain-out shelter and simulated rain events were clearly visible from the temporal dynamics of the volumetric soil water content (vol. SWC) in all depths, which spanned from 3 to 36%. Infiltration of irrigation water was most visible in the upper soil depths (−2 and −5 cm) whereas the lower depths (−20 and −40 cm) were less affected and showed overall more stable vol. SWC values. Variation among replicates showed to be highest in −2 cm and in −40 cm depth, as well as after each applied irrigation event. After the above-mentioned rainfall event on July 5, a short peak was observable in the two deeper soil horizons, most likely due to preferential flow along soil cracks. Except for this peak value in −40 cm, highest differences of vol. SWC during the measurement period were observed at −2 cm, with values ranging from 7 to 36%.

### Extraction Efficiency of Cryogenic Vacuum Extraction and Centrifugation

Generally, the water recovery rate of cryogenic vacuum extraction was higher compared to that of the centrifugation. In our study, the centrifugation of soils was strongly limited by sampled material and soil water content. Out of 64 samples for centrifugation, only 22 delivered enough liquid water for isotope analysis, i.e., more than 65% samples could not be measured. The limit for centrifugation was a vol. SWC of 7% in the lower soil depths (−20 and −40 cm). In the upper soil depths (−2 and −5 cm) with higher organic content the limit for centrifugation was 13%. However, we often could not obtain enough water for higher vol. SWC values in all depths, either. Generally, more soil material (here >15 ml/35 g) would have been needed to successfully determine the isotopic signature of soil water with centrifugation.

None of the soil samples were found to be organically contaminated (Chem Correct results).

Isotopic signatures could be measured from 92% of the soil water samples cryogenically extracted. Loss of samples was mainly related to difficulties in sample collection in the lower soil horizons (extraction efficiency of 75% in soil depths −20 and −40 cm).

### Performance of *In Situ* and Destructive Isotope Measurements

The mean measurement precision of GP tubes in the medium soil standard (STD_M_: δ^18^O = −9.3 ± 0.07‰; δ^2^H = −65.3 ± 0.32‰) deviated by 0.3‰ and 0.5‰ from the target value for δ^18^O and δ^2^H, respectively. This expresses the average correction applied to values from *in situ* derived soil water measurements to account for drift correction of the laser spectroscope due to fluctuating background conditions. No apparent drift in standard isotope ratios was observed.

The measurement precision of the laser spectroscope in the laboratory, i.e., the precision for the destructive approach, was 0.3 ± 0.4‰ for δ^18^O and 1.2 ± 6.2‰ for δ^2^H, and again no drift was observed.

The accuracy of the cryogenic extraction, tested on spiked replicates from Freiburg field site, showed that mean deviations from the target value were 0.6 ± 0.2‰ for δ^18^O and 3.0 ± 1.2‰ for δ^2^H, respectively. This was less precise than results of our repeated *in situ* soil standard measurements in sand, and laboratory results with GP tubing (data not shown) in the same soil as used in the spike tests.

### Method Comparison

#### Derived Isotopic Signature Values

**FIGURE 4:**
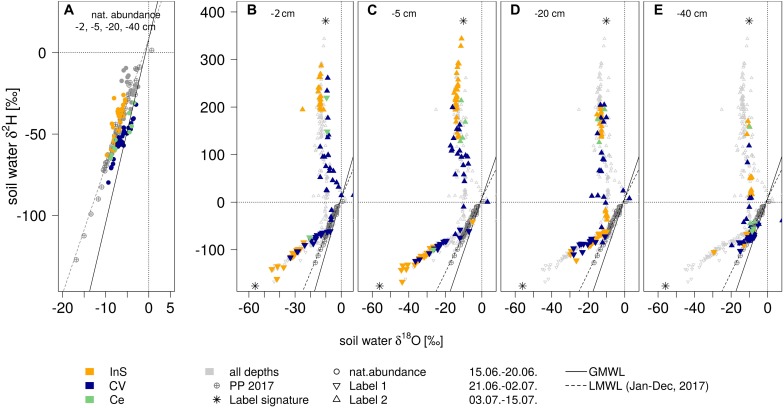
Dual isotopes plots of soil water (δ^18^O vs. δ^2^H): **(A)** before (all soil depths) and **(B–E)** after the irrigation events (per depth) resulting from different extraction methods (*in situ* gas permeable tubing InS, cryogenic vacuum extraction CV and centrifugation Ce, in: yellow, blue, green resp.). Global meteoric water line GMWL and local meteoric water line (LMWL, based on 2017 data). *indicate isotopic signatures of irrigation events.

**FIGURE 5:**
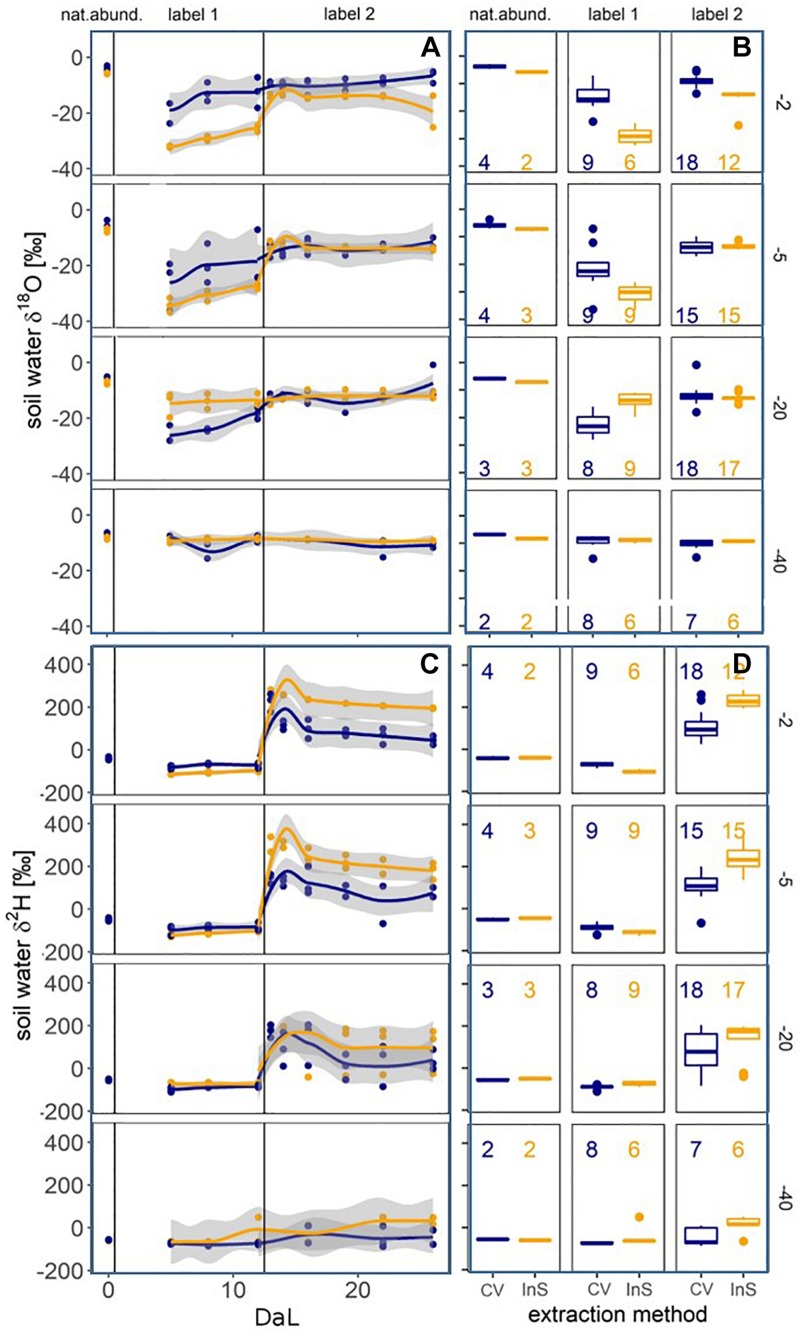
Soil water δ^18^O **(A,B)** and δ^2^H **(C,D)** values measured at −2, −5, −20, and −40 cm depth by cryogenic vacuum extraction (CV, blue) and *in situ* gas permeable tubing (InS, yellow). Left panels **(A,C)**: Evolution over time (Day after labelling, DaL). Right panels **(B,D)**: Boxplots per experimental phase with sample size (at least *n* = 2 per time, depth and method). Methods were considered significantly different if confidence intervals of loess regressions did not overlap.

During the natural abundance measurement phase, mean isotopic signatures of all extraction methods (method centrifugation excluded) for soil water were similar to mean precipitation values (−7.3 ± 4‰ in δ^18^O and −48.3 ± 28‰ in δ^2^H): Mean *in situ* measured values (across soil depths) were −7.1 ± 1‰ for δ^18^O and −48.6 ± 8‰ for δ^2^H. Cryogenic vacuum extraction obtained higher values for δ^18^O with −5.5 ± 2‰, but lower for δ^2^H with −50.1 ± 11‰ (Supplementary Table S1). After irrigation with labelled waters, isotopic signatures of both soil water extraction methods clearly showed that antecedent and newly incoming water mixed (Figure 4). Soil waters, observed after the two labelling pulses, strongly differed in dual isotope space (δ^18^O, δ^2^H). The *in situ* soil water vapor method, generally, showed the strongest deviation of isotopic signatures from preconditions (Figure 5). This effect was most visible in −2 and −5 cm depth and diminished with depths. While the isotopic response was more pronounced for δ^18^O following labelling pulse 1 (label strongly depleted in ^18^O relative to natural abundance), δ^2^H was more affected after labelling pulse 2 (strongly enriched in ^2^H). After labelling pulse 1, the isotopic signature inferred from cryogenic vacuum extraction was higher, with an average difference of 3.4‰ for δ^18^O and 5.0‰ for δ^2^H over all depths (Supplementary Table S2). Significant differences were found for δ^18^O in −2 and −20 cm depth (no overlap of confidence intervals, Figure 5). After labelling pulse 2, mean differences in δ^18^O were minor between methods (0.9‰), but high in δ^2^H with 107.5‰ (with high standard deviations for both methods >260‰). Differences in isotopic signatures were significant for the observations in depths −2 and −5 cm (no overlap, Figure 5). Soil isotopic signature at −40 cm depth was visibly affected by the second labelled irrigation only when it diverged from natural abundance. This could be observed by both methods but was more pronounced for the *in situ* soil water vapor method.

#### Temporal Evolution and Variation of δ^18^O and δ^2^H in Soil Profiles

**FIGURE 6:**
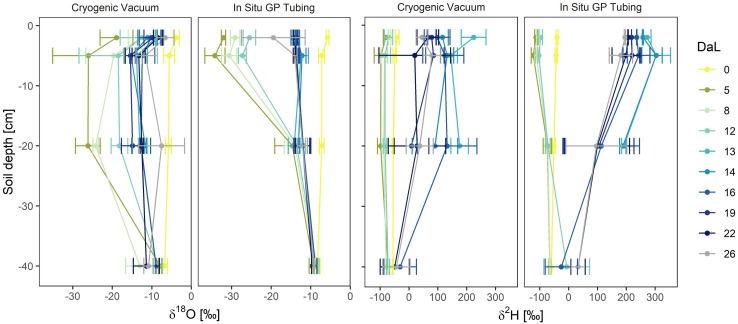
Temporal dynamics of soil water δ^18^O and δ^2^H profiles (Day of Labelling, DaL) obtained by cryogenic vacuum extraction and *in situ* gas permeable (GP) tubing (*n* = 2–3). Mean values ± 1 SD. Labelling pulse 1 was applied in the night after DaL 0, label 2 in the night after DaL 12.

Depth profiles of soil water isotopic signatures obtained from the *in situ* and cryogenic vacuum extraction methods are presented in Figure 6. At initial conditions (DaL = 0), soil water isotope profiles showed a typical exponential shape, with the strongest enrichment of both isotopologues in −2 cm depth. The first labelling pulse substantially decreased mean signatures of δ^18^O and δ^2^H in the topsoil (from DaL 0 to DaL 5). For the *in situ* method in −2 cm depth, we observed a decrease from −5.7 to −32.0‰ (δ^18^O) and from −40.1 to −113.2‰ (δ^2^H). For the cryogenic vacuum extraction, we saw a decrease from −3.8 to −33.1‰ (δ^18^O) and −40.4 to −116.3‰ (δ^2^H). The second labelling pulse increased δ^18^O values of cryogenically extracted soil water from −12.8 to −8.9‰ and increased δ^2^H values from −70.8 to +224.1‰ in −2 cm depth. For the *in situ* method, we found an increase from −25.5 to −13.7‰ for δ^18^O; from −97.0 to +271.5‰ for δ^2^H.

Under natural abundance conditions, average SD in soil water δ^18^O and δ^2^H measured across depths was, generally, in a similar range, with 1.2‰ and 3.5‰, respectively, for the *in situ* soil water vapor method and 1.9‰, and 9.9‰ for cryogenic vacuum extraction (Supplementary Table S1). Irrigation events increased the range of soil water isotopic signatures considerably for both methods and therewith the isotopic differences between these methods.

While the *in situ* soil water vapor method clearly showed the temporal development of the soil water vapor isotopic signature (Supplementary Figure S2) of the same undisturbed soil volume, it has to be taken into account that the cryogenic vacuum extraction method based on destructive sampling at different location captured both the temporal variability and lateral heterogeneity of the soil profile. For the *in situ* method, isotopic differences across soil depths were better distinguishable, especially after the labelling pulses. Rothfuss et al., 2013, observed a memory effect associated with *in situ* GP tubes under saturated conditions (pure water). This was not the case in our experiment, since the soil was never fully saturated, even not directly after irrigation events.

#### Time and Cost Expenses

In terms of effort and costs, each extraction method had its advantages and disadvantages (Table 1). The time required for *in situ* soil sampling in our experiment was approximately 25 h per 100 samples, i.e., on average 15 min per sample, not including calibration and flushing of the system with dry air. Additionally, two entire days were needed for installation of the experimental setup. The equipment needed for the *in situ* soil method is cheaper than for the destructive methods and no material, despite of gas and electricity, is consumed. For the setup and handling of the *in situ* system an experienced person is needed, and further tests are necessary before the system is ready for operational use.

**TABLE 1:** Overview over costs [€] and time [h] needed in this experiment for extraction of water, per 100 samples.

	Destructive		*In situ*
	Cryo. vacuum extraction	Centrifuge	Polyprop.
Equipment	8,000	5,000	1,775
Tubing			14.2^2^
Labor intensity	Intense	Low	Low
Time [h]^1^	60^3^	58.3^3^	25
Running costs^1^	120	610	–
Know-how for setup and handling	Medium	Easy	Medium-difficult

*Not included are the time required for calibration of isotopic signatures, as well as costs for power and gas supply. ^1^per 100 sample, ^2^per soil depth, and ^3^including laser measurement with 6 injections per sample.*

In total, it took 36 min per sample (without calibration) from soil sampling over cryogenic vacuum extraction to isotope analysis in the lab via laser spectroscope.

## Discussion

### Methodological Differences

All extraction methods but centrifugation were able to generate isotopic signatures for soil water. However, some samples from deeper soil depths did not contain enough water for cryogenic vacuum extraction and subsequent liquid isotope analysis (i.e., 25% samples from −20 cm and −40 cm depth). The *in situ* monitoring method worked well for lower soil water contents and under stable environmental conditions (no rain and low rH), which both lowered the risk of condensation in the GP tubing.

We found the strongest impact of labelled water on the soil water in the uppermost soil layer, decreasing isotopic variation (i.e., smaller SDs) with depth, and relatively constant isotopic values in −40 cm depth for both methods throughout the whole experiment (Figures 5, 6). Several studies reported a damping of incoming precipitation signals in the soil profile and a decreasing variability of soil water isotopes with depth, independently from sampling strategies (Orlowski et al., 2016a; Sprenger et al., 2016). They concluded that this observed damping was related to soil water mixing and capillary rise of groundwater. This dampening effect was observed for all extraction methods in all depths and therefore mixing seems to always occur to some degree.

We observed substantial differences between cryogenically extracted and *in situ* soil water vapor measurements, particularly after label 2 which was strongly enriched in ^2^H (Figures 5, 6). A generally lower effect of the labelled water to the on the cryogenically extracted soil water was found.

The fraction of soil water sampled by different extraction methods is currently one of the main questions faced by the ecohydrological research community (Berry et al., 2018; Penna et al., 2018; Sprenger et al., 2018; Orlowski et al., 2019). In comparison to the direct water vapor equilibration method, cryogenic vacuum extraction can likely also access hygroscopic and biologically bound water (Koeniger et al., 2011; Sprenger et al., 2015; Orlowski et al., 2016b). When testing different soil types with varying soil water contents, generally higher δ^18^O and δ^2^H values have been reported for the direct water vapor equilibration method in comparison to cryogenic vacuum extraction (Orlowski et al., 2016b). However, we did not find higher δ^18^O and δ^2^H values for the *in situ* soil water vapor method here.

Some authors (Oerter and Bowen, 2017) could observe systematically higher values (5.7‰ for δ^2^H and 1.1‰ for δ^18^O) between *in situ* GP and cryogenically derived isotopic signatures under natural conditions while others did not observe systematic offsets (Volkmann and Weiler, 2014 and Gaj et al., 2016). Oerter and Bowen (2017) concluded that a physical separation of soil water, related to the characteristics of the soil material (like particle size) occurring in their studied system, was the reason for that difference. The initial water content and the size of particles were found to influence how antecedent water was replaced by subsequent water infiltration events (Gouet-Kaplan et al., 2012). This means that the *in situ* GP method, which always monitored the identical area, could not capture the spatial isotopic heterogeneity of the soil system. The destructive sampling for the cryogenic vacuum extraction, however, included the soil system’s spatial isotopic variation. By adding labeled water with an isotopic signature which strongly differed from natural abundance, we highly increased the isotopic gradients in the soil. In addition, even though the amount of added water was high for a rainfall event, it did not completely saturate the soil. This further increased the spatial heterogeneity of water isotopic signatures in the soil. Hence, we could observe a high isotopic variation in our soil which likely caused the offset of 107.5‰ in δ^2^H between cryogenic vacuum extraction and *in situ* derived δ^2^H, linked to the (spatially different) sampling of the methods. The *in situ* soil water vapor method always sampled the identical soil area while the soil samples for the cryogenic vacuum extraction were taken from different areas and thus covered a wider lateral range.

### Strength and Limitations of Soil Water Vapor *In Situ* Method

A main constrain for the *in situ* soil water vapor method is the access to power and gas supply in the field, as well as the possibility to set up the equipment in an, ideally air conditioned, shelter. One further crucial point of the *in situ* soil water vapor method is the installation of the sampling tubes in the field. It can substantially alter the soil’s natural structure and therewith impact water flow pathways, especially in highly heterogeneous soils, as the one of our field site, with distinct horizons from fluvial depositions. Therefore, this destructive impact should be kept minimal during installation. Installations are recommended ahead of time (>60 days) to allow soil to settle and vegetation to fully regrow before starting *in situ* measurements. A potential source of error can arise from condensations effect. We impeded condensation effects by establishment of a flushing routine which was checked regularly and if necessary adapted based on the actual requirements. A dilution routine right after the sampling point (Quade et al., 2019) or a temperature control system [i.e., a heating system, Oerter and Bowen (2017)] would be an ideal countermeasure against condensation if the experimental setup allows it, in particular for measurements under wet and cool ambient conditions. It should be further monitored how soil conditions, sampling procedure and timeframe affect the measurement results. Rothfuss et al. (2015) reported no impact on the isotopic signature of water vapor after sampling over long periods and high flow rates under laboratory conditions (soil type: FH31 sand). Similarly, we could not observe an impact of sampling, i.e., an isotopic enrichment of our standard vessels, either. Moreover, sampling was not limited by low soil water contents and could repeatedly take place at the identical soil location. Strengths and limitations of the *in situ* soil water vapor method are discussed in more detail in Quade et al. (2018, 2019).

### How to Choose Which Method to Use?

In terms of effort and costs, each extraction method had its advantages and disadvantages (Table 1). While the centrifugation is less labor-intensive than the other two extraction methods, it is relatively expensive and limited in its application for soil samples, regarding the soil water content and soil type (Orlowski et al., 2016b).

The *in situ* method was, generally, the fastest method (i.e., 25 h for 100 samples) to derive isotopic signature of soil water and the cheapest regarding the initial equipment. It was also relatively cheap during operation since it only consumed power and gas (i.e., laser spectroscope related consumables which are needed for all extraction methods). The initial investments of time and expertise for the *in situ* method are compensated by shorter sampling times and lower operating costs, in particular if the system is automated.

The *in situ* soil water vapor method had a high measurement precision of 0.3‰ for δ^18^O and 0.2‰ for δ^2^H. With a deviation of −0.6 ± 0.2‰ for δ^18^O and +3.0 ± 1.2‰ for δ^2^H from the target value (spike test), our system for cryogenic vacuum extraction did not meet the criteria of an acceptable performance (±0.2‰ for δ^18^O and ± 2‰ for δ^2^H, Wassenaar et al., 2012; Orlowski et al., 2016b). The application of cryogenic vacuum extraction is currently strongly discussed in ecohydrological research and associated with strong varying results dependent on soil texture, soil water contents and laboratory procedure (Orlowski et al., 2013, 2016b, 2018). In the worldwide comparison of cryogenic vacuum extraction systems, our system delivered a relatively high precision (Orlowski et al., 2018). The measurement with the laser spectroscope further decreased the measurement precision (laser spectroscope: 0.3 ± 0.4‰ for δ^18^O and 1.2 ± 6.2‰ for δ^2^H). The heterogeneity and higher variation of cryogenic vacuum extraction (Figure 6), associated with the destructive sampling bias and the extraction procedure, led to a wide range of observed isotopic signatures. This high variation can impede the use of isotopic signatures to distinguish between different soil depths and, hence, to trace water flow pathways. By sampling the identical soil area, the *in situ* soil water vapor method can exclude the bias of destructive sampling and decrease sampling related heterogeneity. This is of significant advantage when applying isotopically labelled waters in heterogeneous soil systems. Labeling approaches have been widely applied in ecohydrological studies [e.g., Volkmann et al. (2016a), Quade et al. (2018)] and are a powerful tool to trace water fluxes. They allow a statistically robust distinction between different water sources (Seeger and Weiler, 2016), which is important for statistical mixing source water models [e.g., *SIAR* by Parnell et al. (2010)]. By sampling the identical soil area and avoiding sampling related heterogeneity, the *in situ* soil water vapor method will further increase the accuracy of these models. To capture the vertical as well as the natural lateral isotopic heterogeneity of a system and their temporal dynamics, several soil profiles with *in situ* GP tubes could be laterally distributed to obtain 3D high temporal resolution soil isotopic profiles.

The choice of method should not only consider the prevailing soil conditions (Orlowski et al., 2016b) but also the experimental design and goal, i.e., which spatial and/or temporal resolution of processes should be captured (Dubbert and Werner, 2019). The two different approaches (destructive vs. *in situ*) aim at different spatial and temporal scales and resolutions. In this study, the isotopic signatures obtained by cryogenic vacuum extraction only represented 10 cm^3^ of the soil volume. Our *in situ* soil water vapor method could sample 42 cm^3^ under near-saturated, and even up to 526 cm^3^ under dry conditions (Quade et al., 2019). The destructive approach only delivers data for one specific point in time, the time of destructive sampling. The *in situ* method, however, can resolve processes occurring at very small time scales. It is only temporally limited in the availability of the laser spectroscope for measurements.

So far, the *in situ* method has been applied mainly at smaller spatial scales, and more research is needed to also implement this technique at wider spatial scales. Longer tubing could be applied (Gangi et al., 2015) to sample larger soil volumes and increase the spatial extent. But longer tubing increases the risk of dry air leaving the gas permeable tubing, in particular under dry conditions (Quade et al., 2019). To assess soil water processes and soil-plant interactions adequately and identify underlying mechanisms, high temporal resolution observations for both soil and plant related processes are pivotal. More research is needed to also implement the *in situ* soil water vapor method on larger spatial scales. We see a very promising tool in the *in situ* soil water vapor method to capture temporal developments and spatial variability of soil water and ecohydrological processes, in particular in combination with *in situ* xylem or transpiration monitoring techniques.

## Data Availability Statement

The datasets generated for this study are available on request to the corresponding author.

## Author Contributions

AK wrote the manuscript. SP helped writing the manuscript. AK, MD, YR, and NO conceived the study design. AK and MD planned the experiment. SP, AK, and AD set up the experiment and collected the data. SP and AK analyzed the data. All authors reviewed the manuscript.

## Conflict of Interest

The authors declare that the research was conducted in the absence of any commercial or financial relationships that could be construed as a potential conflict of interest.
