# Supplementary material for: Water Stable Isotopes in Ecohydrological Field Research: Comparison Between In Situ and Destructive Monitoring Methods to Determine Soil Water Isotopic Signatures
Source: Front Plant Sci. 2020 Apr 14;11:387. doi: 10.3389/fpls.2020.00387 (PMC7171290; doi:10.3389/fpls.2020.00387)
Supplement: Supplementary file 1 [file Table_1.DOCX]

Supplementary Material


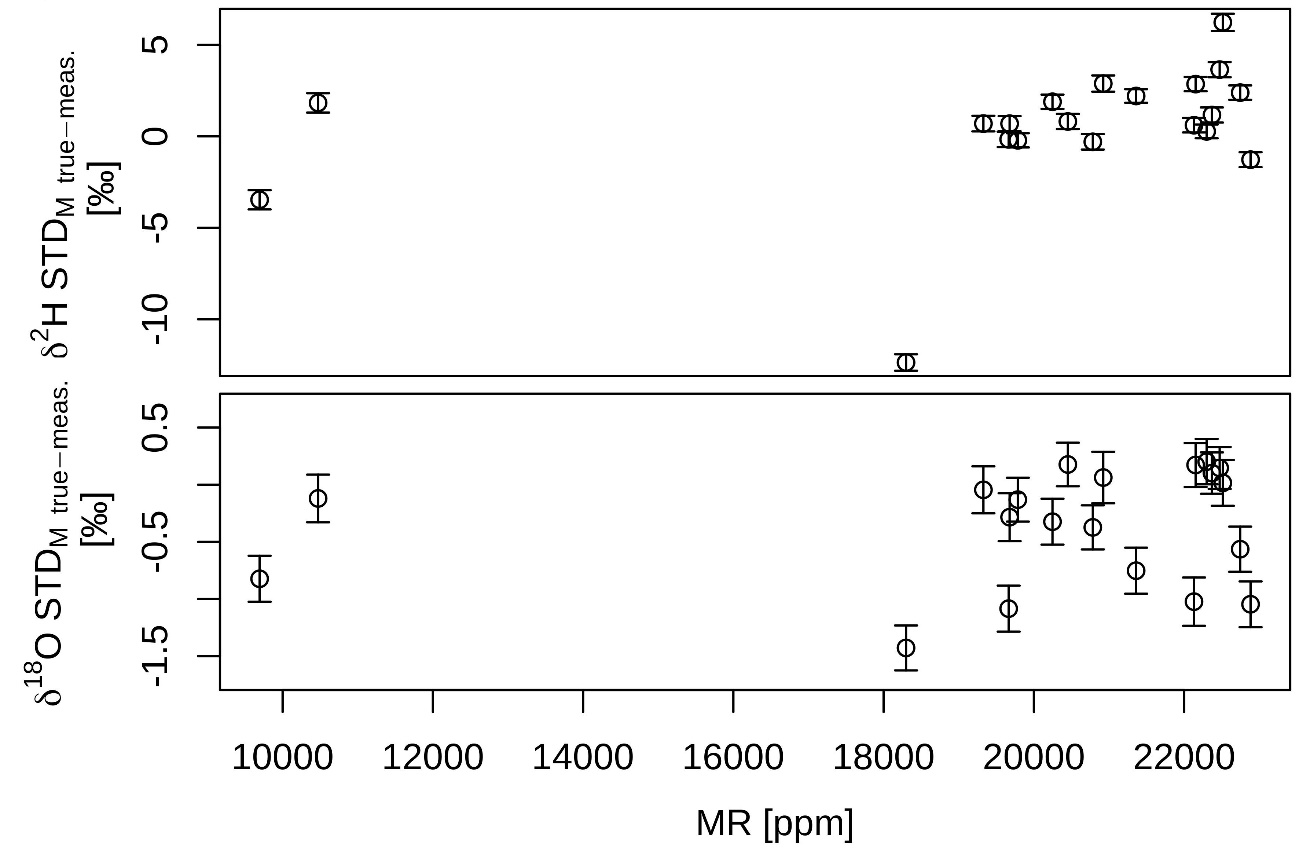


**Supplementary Figure 1:** Relation between soil water vapor mixing ratio (MR, in ppm) and deviation of measured δs_l value from the true δs_l value of soil standard vessel STD_M [‰] (Top panel: δ^2^H vs. MR; bottom panel: δ^18^O vs. MR). Mean ± 1 sd. Derived equations from linear regressions were not significant for both isotopologues (δ^2^H: p = 0.15, R^2^ = 0.11; δ^18^O: p = 0.8, R^2^ = ‑0.04).

Supplementary Table 1: Mean soil water δ^18^O and δ^2^H values ±1 sd [‰] per experimental phase (natural abundance, label 1, label 2) and type of extraction method (cryogenic vacuum extraction CV, in situ polypropylene tubing InS). Per soil depth (-2, -5, -20, and -40 cm) and averaged over all depths (last four columns).

| soil depth  [cm] | phase | method | δ^18^O  [‰] | ±sd  [‰] | δ^2^H  [‰] | ±sd  [‰] | δ^18^O  [‰] | ±sd  [‰] | δ^2^H  [‰] | ± sd  [‰] |
| --- | --- | --- | --- | --- | --- | --- | --- | --- | --- | --- |
| -2 | nat. abund. | CV | -3.8 | 0.8 | -40.5 | 6.2 |  |  |  |  |
| -5 | nat. abund. | CV | -5.6 | 1.4 | -49.6 | 6.3 |  |  |  |  |
| -20 | nat. abund. | CV | -5.8 | 0.8 | -54.4 | 4.1 |  |  |  |  |
| -40 | nat. abund. | CV | -6.8 | 0.7 | -55.8 | 1.8 | -5.5 | 1.9 | -50.1 | 9.9 |
| -2 | nat. abund. | InS | -5.7 | 0.3 | -40.1 | 3.8 |  |  |  |  |
| -5 | nat. abund. | InS | -7.2 | 0.7 | -43.1 | 4.1 |  |  |  |  |
| -20 | nat. abund. | InS | -7.2 | 0.6 | -51.0 | 4.9 |  |  |  |  |
| -40 | nat. abund. | InS | -8.3 | 0.7 | -60.1 | 1.3 | -7.1 | 1.2 | -48.6 | 7.5 |
| -2 | label 1 | CV | -14.7 | 7.7 | -72.6 | 17.2 |  |  |  |  |
| -5 | label 1 | CV | -21.4 | 15.1 | -88.9 | 32.6 |  |  |  |  |
| -20 | label 1 | CV | -22.9 | 3.8 | -89.9 | 12.5 |  |  |  |  |
| -40 | label 1 | CV | -9.8 | 3.8 | -74.8 | 10.1 | -17.2 | 17.8 | -81.6 | 40.2 |
| -2 | label 1 | InS | -28.9 | 2.1 | -105.4 | 9.6 |  |  |  |  |
| -5 | label 1 | InS | -30.7 | 3.4 | -112.6 | 9.0 |  |  |  |  |
| -20 | label 1 | InS | -14.0 | 5.6 | -72.4 | 18.8 |  |  |  |  |
| -40 | label 1 | InS | -8.9 | 1.5 | -64.4 | 4.3 | -20.6 | 7.1 | -88.7 | 23.3 |
| -2 | label 2 | CV | -9.2 | 5.1 | 104.5 | 79.4 |  |  |  |  |
| -5 | label 2 | CV | -13.7 | 5.5 | 97.5 | 152.2 |  |  |  |  |
| -20 | label 2 | CV | -13.0 | 7.1 | 86.8 | 183.1 |  |  |  |  |
| -40 | label 2 | CV | -10.4 | 3.5 | -41.3 | 89.2 | -11.6 | 10.9 | 61.9 | 266.3 |
| -2 | label 2 | InS | -14.8 | 8.2 | 230.7 | 14.5 |  |  |  |  |
| -5 | label 2 | InS | -13.3 | 2.6 | 249.7 | 101.2 |  |  |  |  |
| -20 | label 2 | InS | -12.7 | 3.9 | 164.4 | 237.0 |  |  |  |  |
| -40 | label 2 | InS | -9.2 | 0.4 | 32.5 | 41.6 | -12.5 | 9.4 | 169.3 | 261.5 |

Supplementary Table 2: Mean δ^18^O and δ^2^H differences [‰] between soil water extraction methods (cryogenic vacuum extraction CV, in situ polypropylene InS) per experimental phase (natural abundance, label 1, label 2). Per soil depth (-2, -5, -20, and -40 cm) and averaged over all depths (last two columns).

| soil depth [cm] | phase | δ^18^O [‰] | δ^2^H [‰] | δ^18^O [‰] | δ^2^H [‰] |
| --- | --- | --- | --- | --- | --- |
| -2 | nat. abund. | 1.9 | -0.4 |  |  |
| -5 | nat. abund. | 1.6 | -6.5 |  |  |
| -20 | nat. abund. | 1.4 | -3.4 |  |  |
| -40 | nat. abund. | 1.5 | 4.3 | 1.6 | -1.5 |
| -2 | label 1 | 14.2 | 32.8 |  |  |
| -5 | label 1 | 9.3 | 23.7 |  |  |
| -20 | label 1 | -8.8 | -17.4 |  |  |
| -40 | label 1 | -0.9 | -10.4 | 3.4 | 7.2 |
| -2 | label 2 | 5.6 | -126.2 |  |  |
| -5 | label 2 | -0.4 | -152.2 |  |  |
| -20 | label 2 | -0.3 | -77.6 |  |  |
| -40 | label 2 | -1.2 | -73.8 | 0.9 | 107.5 |


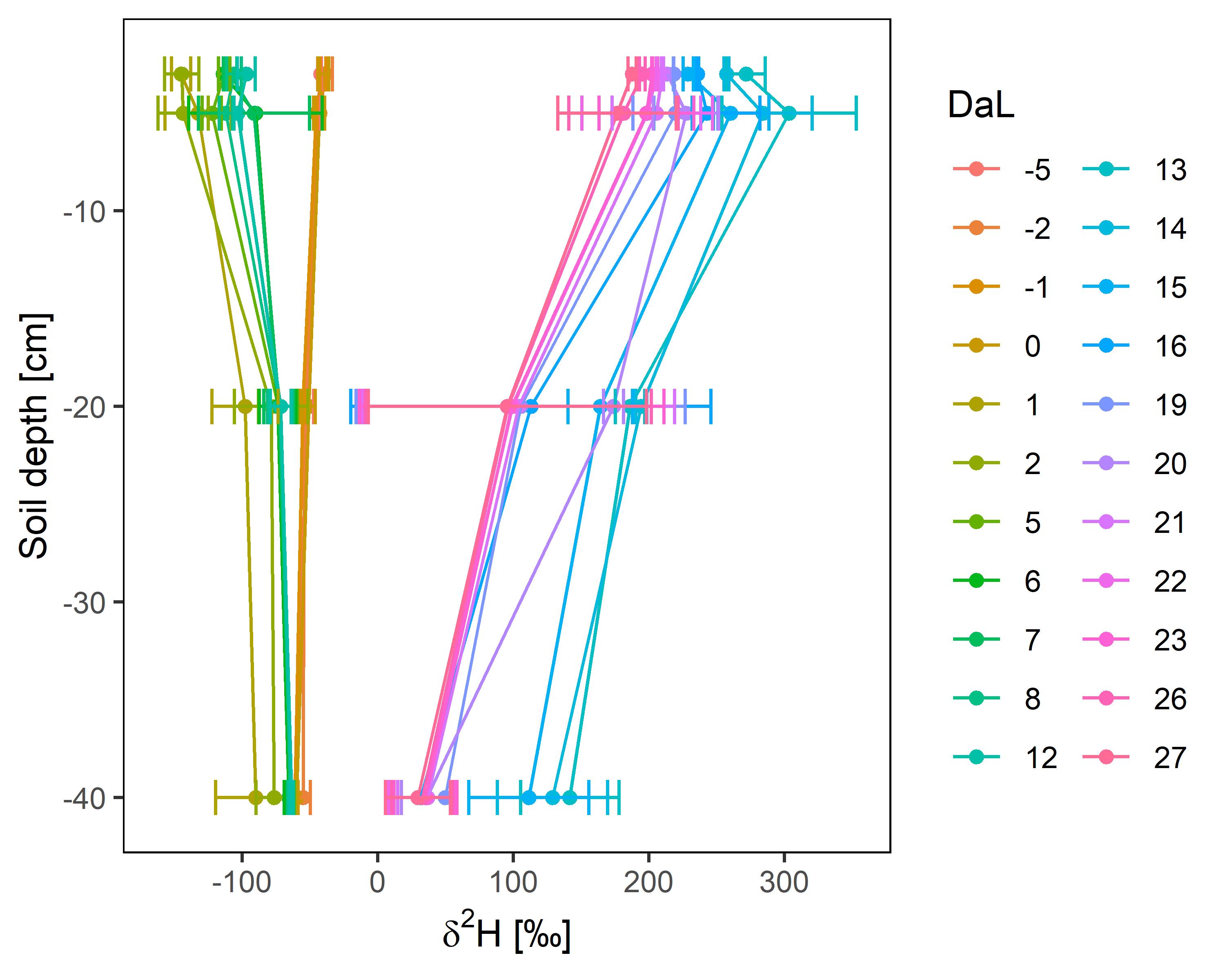


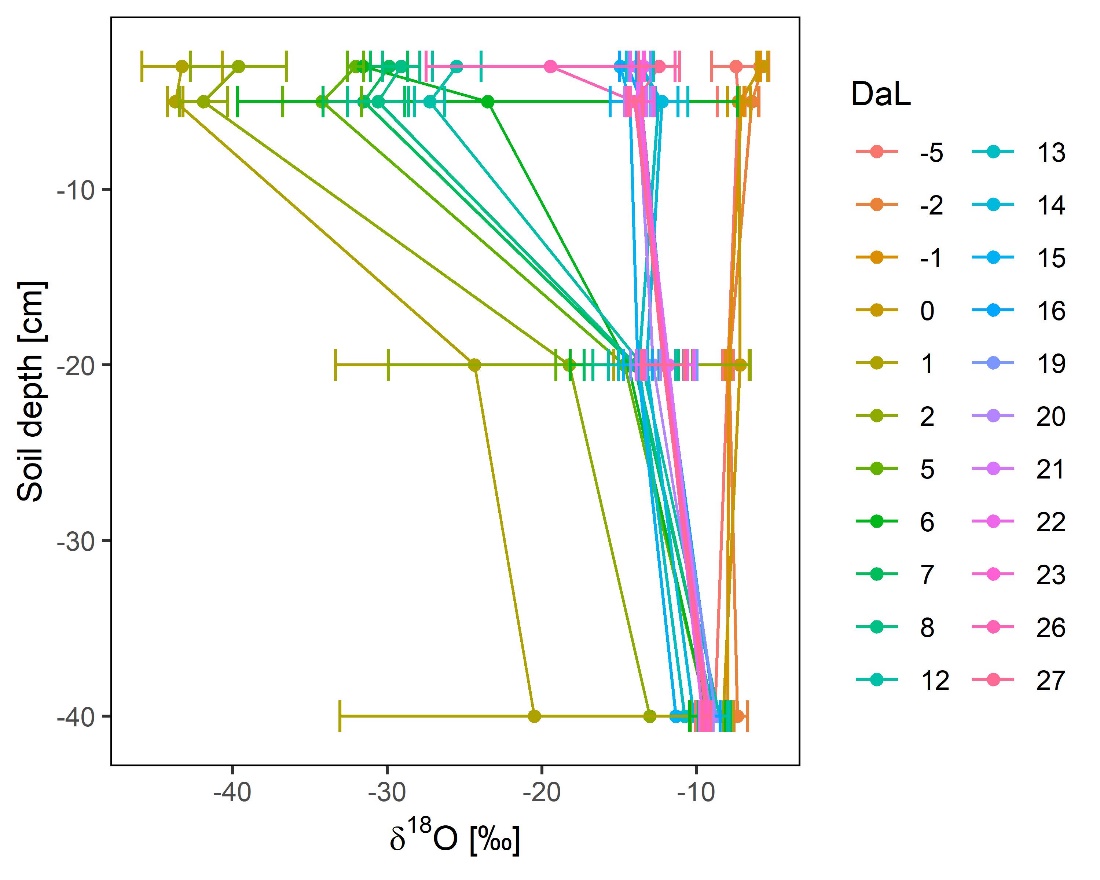


Supplementary Figure 2: Temporal dynamics (DaL: Day after Labeling) of soil water isotopic signature profiles (Top panel: δ^18^O; bottom panel: δ^2^H, [%]) derived with the in situ soil water vapor extraction (InS). Top: δ^18^O. Bottom: δ^2^H. Error bars indicate 1 sd.
